# Preparationof Konjac Glucomannan-Based Composite Films Loaded with Hydroxypropyl-β-Cyclodextrin/Hesperetin Inclusion Complex and Their Preservation Effect on Strawberries

**DOI:** 10.3390/foods15132356

**Published:** 2026-07-02

**Authors:** Guangsen Li, Junwei Zhang, Youxiang Wu, Xiang Wang, Xin Lei, Hanyu Wei, Zhiwen Hu, Qibiao Weng, Qinhua Zhang, Chengrong Wen, Yilan Sun, Qian Ning

**Affiliations:** 1 College of Food Science, Fujian Agriculture and Forestry University, Fuzhou 350002, China; 2 Fujian Provincial Key Laboratory of Eel Breeding and Processing, Fuzhou 350208, China; 3 College of Food Science, Jinshan College of Fujian Agriculture and Forestry University, Fuzhou 350002, China; 4 School of Food Science and Technology, Dalian Polytechnic University, Dalian 116034, China

**Keywords:** konjac glucomannan, hesperetin, hydroxypropyl-β-cyclodextrin, active packaging, strawberry preservation

## Abstract

A KGM/CMC/PVA film loaded with a hydroxypropy-β-cyclodextrin/hesperetin (HP-β-CD/HES) inclusion complex was developed for strawberry preservation. The inclusion complex showed an encapsulation efficiency of 70.41 ± 4.01%. Loading the inclusion complex enhanced the barrier and bioactive properties of the films. At the better loading level (KCP-HH3, 1.5%), the film provided nearly complete UV shielding at 200–350 nm and showed a transmittance of approximately 21% at 800 nm. Relative to KCP, KCP-HH3 exhibited lower water vapor permeability (0.69 ± 0.06 g·mm/(m^2^·h·kPa)) and oxygen permeability (0.066 ± 0.006 g·mm/(m^2^·h·kPa)). The cumulative HES release from KCP-HH3 reached 82.43 ± 2.19% after 7 days at 98% relative humidity. KCP-HH3 scavenged 78.19 ± 3.71% of DPPH radicals and 88.89 ± 4.30% of ABTS radicals. It also inhibited Escherichia coli (91.18 ± 2.99%), Staphylococcus aureus (79.21 ± 3.45%), and Aspergillus niger (92.85 ± 2.75%). This highest-loading formulation delivered the best overall strawberry-preservation performance. After 12 days of storage, strawberries packaged with KCP-HH3 showed higher firmness (0.89 ± 0.04 N) and total soluble solids (7.96 ± 0.21%), as well as lower weight loss (14.30 ± 0.40%), than the polyethylene control, which showed a firmness of 0.42 ± 0.05 N, total soluble solids of 6.22 ± 0.20%, and weight loss of 16.36 ± 0.66%. These results support the potential of HP-β-CD/HES-loaded KCP films as biodegradable active packaging for fruit preservation.

## 1. Introduction

Food safety is closely associated with human health, and food packaging plays a crucial role in maintaining food safety, minimizing external contamination, and extending shelf life [1]. With the growing demand for high-quality preservation of perishable foods, conventional packaging systems are often insufficient to meet practical storage requirements. In contrast, active packaging can regulate the microenvironment surrounding food through the incorporation of functional agents and has therefore attracted considerable attention in the field of food packaging. Strawberries are highly perishable fruits because of their high moisture content, soft tissue structure, active respiration, and susceptibility to microbial spoilage during storage and distribution [2]. Their quality deterioration is usually associated with water loss, softening, oxidative reactions, changes in soluble solids and acidity, and visible fungal decay [3]. Therefore, biodegradable active packaging films that combine barrier protection with antioxidant and antimicrobial functions are of practical significance for delaying strawberry quality deterioration [4].

Konjac glucomannan (KGM), a natural polysaccharide, has excellent film-forming ability and biodegradability, making it a promising material for bio-based packaging films [5]. However, pure KGM films generally exhibit high hydrophilicity, poor mechanical strength, and limited functionality, which restrict their practical application in active packaging and necessitate modification through blending with other film-forming materials [6]. Among these materials, carboxymethyl cellulose (CMC) and polyvinyl alcohol (PVA) have been widely used to improve the mechanical performance and barrier properties of polysaccharide-based films. CMC can improve the continuity and compatibility of polysaccharide networks through intermolecular interactions, while PVA provides good film-forming ability, flexibility, and structural stability [7]. Therefore, the combination of KGM, CMC, and PVA provides an effective strategy for constructing KGM-based active packaging systems with improved mechanical integrity and barrier performance.

Hesperetin (HES) is a natural flavanone obtained from hesperidin-rich citrus-processing by-products, particularly citrus peels. It exhibits antioxidant activity and antimicrobial potential against Gram-positive and Gram-negative bacteria, as well as several fungi, including *Aspergillus* spp., making it a promising functional additive for active packaging films. However, its poor water solubility can lead to uneven dispersion and low utilization efficiency in hydrophilic film-forming systems [8,9]. Hydroxypropyl-β-cyclodextrin (HP-β-CD), owing to its hydrophobic inner cavity and hydrophilic outer surface, can form host-guest inclusion complexes with hydrophobic compounds, thereby improving their dispersibility and stability [10]. Previous studies have demonstrated that cyclodextrin encapsulation can enhance the solubility, bioactivity, and delivery efficiency of HES [11]. Therefore, incorporation of an HP-β-CD/HES inclusion complex improves the compatibility and functional performance of HES in hydrophilic KGM-based composite films. Nevertheless, reports on the application of HES in bio-based composite films, particularly for fruit preservation, remain limited. Therefore, in the present study, an HP-β-CD/HES inclusion complex was incorporated into a KGM/CMC/PVA composite film matrix to develop a KGM-based active packaging film. Its microstructure, physicochemical properties, functional performance, and preservation effect on strawberries were systematically investigated to provide experimental support for the application of active packaging in fruit preservation.

## 2. Materials and Methods

### 2.1. Materials

Konjac glucomannan was purchased from Hubei Yizhi Konjac Biotechnology Co., Ltd. (Yichang, China); polyvinyl alcohol 1799 was purchased from Shanghai Macklin Biochemical Co., Ltd. (Shanghai, China); carboxymethyl cellulose, hydroxypropyl-β-cyclodextrin, glycerol, citric acid, hesperetin, absolute ethanol, potassium persulfate, DPPH, and ABTS were purchased from Shanghai Aladdin Biochemical Technology Co., Ltd. (Shanghai, China) and *Escherichia coli* and *Staphylococcus aureus* were provided by the Laboratory of the College of Food Science, Fujian Agriculture and Forestry University (Fuzhou, China). *Aspergillus niger* was kindly provided by the Food Science Laboratory of Dalian Polytechnic University (Da lian, China), and fresh ‘Hongyan’ strawberries at commercial maturity were purchased from a Yonghui Supermarket in Fuzhou, China, and transported to the laboratory within 0.5 h. Strawberries with uniform size, similar color and maturity, and without visible mechanical damage, disease symptoms, or fungal infection were selected for the preservation experiment.

### 2.2. Preparation of HP-β-CD/HES Inclusion Complex

The HP-β-CD/HES inclusion complex was prepared based on a previously reported co-evaporation method with slight modifications [1]. Briefly, the inclusion complex was obtained by a co-evaporation method at a HES:HP-β-CD molar ratio of 1:2. HES (0.5 g) was first dissolved in 50 mL of absolute ethanol, and HP-β-CD (5.102 g) was dissolved in 10 mL of distilled water to prepare an aqueous HP-β-CD solution. The HES ethanol solution was then slowly added to the HP-β-CD solution under continuous stirring at room temperature. The mixture was stirred at 30 °C for 24 h and subsequently cooled while stirring to allow gradual solvent evaporation. The resulting product was dried in a forced-air oven at 40 °C to constant weight, ground for 20 min to obtain a homogeneous powder, and stored in a desiccator until further use.

### 2.3. Preparation of Composite Films Loaded with HP-β-CD/HES Inclusion Complex

Composite films containing the HP-β-CD/HES inclusion complex were prepared by solution casting. Briefly, KGM, CMC, and PVA solutions were prepared at the specified concentrations. The KGM and CMC concentrations were both set at 0.5% (*w*/*v*) based on preliminary experiments. The KGM/CMC mixed solution and PVA solution were then mixed at a volume ratio of 1:1. Glycerol, citric acid, and different amounts of the HP-β-CD/HES inclusion complex were sequentially added according to the designed formulations under heating and continuous stirring. After further stirring for 1 h, a homogeneous film-forming solution was obtained. The solution was then ultrasonicated for 10 min to eliminate air bubbles, evenly poured into Petri dishes, and dried in a forced-air oven at 40 °C for 24 h. After drying, the films were peeled off and collected. According to the amount of inclusion complex added, the films were designated as KCP-HH1, KCP-HH2, and KCP-HH3, respectively. The detailed compositions and sample codes of the prepared composite films are summarized in Table 1.

### 2.4. Determination of Encapsulation Efficiency (EE)

The reaction medium initially contained approximately 83.3% (*v*/*v*) ethanol, based on mixing 50 mL of absolute ethanol with 10 mL of aqueous HP-β-CD solution. The reaction dispersion was centrifuged at 3000 rpm for 10 min using a high-speed centrifuge (TDL-50, Shanghai Anting Scientific Instrument Factory, Shanghai, China). The amount of free HES in the centrifuged supernatant of the HP-β-CD/HES inclusion complex was determined by UV spectrophotometry (UV-2600, Shimadzu Corporation, Kyoto, Japan). HES standard solutions at different concentrations were prepared in ethanol, and their absorbance was measured at 286 nm to generate a calibration curve. The regression equation was *y* = 0.0339x + 0.0777 (*R^2^* = 0.9923). The absorbance of the centrifuged supernatant was then measured under the same conditions, and the amount of free HES was determined according to the calibration curve. The encapsulation efficiency (*EE*) was calculated as follows:

(1) $$EE(\%)=\frac{m_{t}-m_{f}}{m_{t}}\times 100$$

where $m_{t}$ is the total amount of HES and $m_{f}$ is the amount of free HES.

### 2.5. Release Behavior of HES from Composite Films

The humidity-responsive release of HES from the HP-β-CD/HES-loaded composite films was evaluated based on the procedure described by Qu et al. [12], with minor modifications. Briefly, three controlled relative humidity (RH) environments, representing low, intermediate, and high humidity conditions, were established in sealed desiccators using saturated K_2_CO_3_, NaCl, and K_2_SO_4_ solutions, corresponding to 43%, 75%, and 98% RH, respectively. Film samples with the same mass were placed in each RH chamber and stored for 7 days. At predetermined 24 h intervals, the samples were withdrawn and immersed in 95% ethanol, followed by ultrasonication for 30 min to extract the unreleased HES retained in the film matrix. The absorbance of the extract was measured at 286 nm using a UV–vis spectrophotometer (UV-2600, Shimadzu Corporation, Kyoto, Japan), and the residual HES content was determined from a pre-established calibration curve. The release rate of HES was calculated according to the following equation:

(2) $$R\;\left(\%\right)=\frac{M_{0}-M_{t}}{M_{0}}\times 100$$

where $M_{0}$ denotes the initial HES loading in the composite film (mg), and $M_{t}$ represents the residual HES extracted from the composite film at time *t* (mg).

### 2.6. Zeta Potential

The zeta potentials of HES, HP-β-CD, and the HP-β-CD/HES inclusion complex were measured according to a previously reported method with slight modifications [1]. Briefly, 20 mg of each sample was separately dispersed in 10 mL of deionized water and ultrasonicated for 10 min to obtain uniform dispersions. The zeta potential of each dispersion was then measured at 25 °C using a particle size and zeta potential analyzer (Litesizer 500, Anton Paar GmbH, Graz, Austria).

### 2.7. Scanning Electron Microscopy (SEM)

The surface and cross-section of the films were observed by scanning electron microscopy (SEM) using a scanning electron microscope (NOVA Nano SEM 230, FEI Company, Hillsboro, OR, USA). Prior to observation, the film samples and the cryo-fractured cross-sectional samples obtained after liquid nitrogen treatment were mounted on conductive adhesive and sputter-coated with gold. The SEM accelerating voltage was set at 15 kV.

### 2.8. Fourier Transform Infrared Spectroscopy (FT-IR)

The film samples were scanned using a Fourier transform infrared spectrometer (Thermo Scientific iN10, Thermo Fisher Scientific, Waltham, MA, USA) in ATR mode with a resolution of 4 cm^−1^ over the range of 400–4000 cm^−1^, and 32 scans were collected for each sample.

### 2.9. X-Ray Diffraction (XRD)

The film samples were analyzed using an X-ray diffractometer (Rigaku SmartLab SE, Rigaku Corporation, Tokyo, Japan) over a scanning range of 10–80° (2θ) at a scanning rate of 5°/min.

### 2.10. Optical Properties

The transmittance and UV-blocking properties of the films were measured in the range of 200–800 nm using a UV spectrophotometer (UV-2600, Shimadzu Corporation, Kyoto, Japan).

### 2.11. Thermo-Gravimetric (TG)

The thermal stability of the films was analyzed using a simultaneous thermal analyzer (NETZSCH STA 449 F5, NETZSCH-Gerätebau GmbH, Selb, Germany) under a nitrogen atmosphere. The samples were heated from 30 to 600 °C at a heating rate of 10 °C/min.

### 2.12. Thickness

The film thickness was measured using a digital micrometer (211-101F, Guilin Guanglu Measuring Instrument Co., Ltd., Guilin, China; resolution: 0.001 mm). For each film formulation, five replicate measurements were performed. The thickness was measured at five randomly selected positions on each film sample, and the mean value was recorded as the film thickness. The mean thickness was used in the calculations of tensile strength, water vapor permeability, and oxygen permeability.

### 2.13. Mechanical Properties

The mechanical properties were evaluated using a tensile testing machine (WDW-5, Changchun Xinte Testing Machine Co., Ltd., Changchun, China) according to ASTM standard method D882-09 [13]. The films were cut into rectangular strips (1 × 5 cm). The tensile speed was set at 10 mm/min. Each sample was measured in five parallel replicates. Tensile strength (TS) and elongation at break (EAB) were calculated using the following equations:

(3) $$\mathrm{TS}\;(\mathrm{MPa})=\frac{F}{W\times T}$$

(4) $$EAB\;(\%)=\frac{L-L_{0}}{L_{0}}$$

where *F* is the maximum force applied to the film (N), *W* and *T* are the width and thickness of the film (mm), respectively, and *L* and *L*_0_ are the extended length and initial length of the film (mm), respectively.

### 2.14. Water Vapor Permeability (WVP)

Water vapor permeability (WVP) was determined by the gravimetric method [14]. The test film was placed over a weighing bottle containing 3 g of anhydrous CaCl_2_ and tightly sealed with a rubber band to ensure close contact between the film and the bottle mouth. The bottle was then placed in a desiccator containing saturated NaCl solution (25 °C, 75% RH). The mass change of the bottle was recorded at regular intervals. Each sample was measured in triplicate. WVP was calculated as follows:

(5) $$WVP(g\cdot mm/m^{2}\cdot h\cdot kPa)=\frac{d\times \Delta G}{S\times \Delta P\times \Delta t}$$

where *d* is the film thickness (mm), Δ*G* is the mass change of the bottle during the test interval (g), *S* is the area of the bottle mouth (m^2^), Δ*P* is the water-vapor partial-pressure difference, and Δ*t* is the test time (h).

### 2.15. Oxygen Permeability (OP)

The oxygen permeability (OP) of the films was determined by the deoxygenation absorption method [15]. Briefly, 1 g of activated carbon, 1.5 g of sodium chloride, and 0.5 g of iron powder were accurately weighed into a weighing bottle, which was then sealed with the test film to ensure a tight, leak-free closure. The sealed weighing bottle was placed in a constant-temperature and constant-humidity desiccator and equilibrated at 25 °C and 75% relative humidity. Each sample was measured in triplicate. The oxygen permeability of the film was calculated from the mass change of the weighing bottle within a certain time interval according to the following equation:

(6) $$OP(g\cdot mm/m^{2}\cdot h\cdot kPa)=\frac{d\times \Delta m}{A\times t\times P}$$

where Δ*m* is the mass change of the weighing bottle during time t (g), *d* is the film thickness (mm), *A* is the effective oxygen permeation area of the film (m^2^), *t* is the test time (h), and *P* is the saturated vapor pressure at the test temperature (kPa).

### 2.16. Antioxidant Activity

The antioxidant activity of the composite films was evaluated using DPPH and ABTS radical-scavenging assays. For the DPPH assay, 25 mg of the composite film was dissolved in 10 mL of DPPH solution (0.1 mM) prepared in anhydrous ethanol, mixed thoroughly, and allowed to react in the dark for 1 h. The absorbance was then measured at 517 nm using a UV–visible spectrophotometer (UV-2600, Shimadzu Corporation, Kyoto, Japan).

For the ABTS assay, equal volumes of 7 mM ABTS solution, prepared in deionized water, and 4.9 mM potassium persulfate solution, prepared in deionized water, were mixed to prepare the ABTS radical solution. The mixture was kept in the dark at room temperature for 12 h to allow for complete reaction. Subsequently, 25 mg of the composite film was dissolved in 10 mL of the ABTS radical solution, mixed thoroughly, and reacted for 10 min. The absorbance was then measured at 734 nm using a UV–visible spectrophotometer (UV-2600, Shimadzu Corporation, Kyoto, Japan).

The DPPH and ABTS radical-scavenging activities were calculated as follows:

(7) $$\mathrm{DPPH}\;\mathrm{radical}\;\mathrm{scavenging}\;\mathrm{rate}\;(\%)=\frac{A_{0}-A_{1}}{A_{0}}\times 100$$

(8) $$\mathrm{ABTS}\;\mathrm{radical}\;\mathrm{scavenging}\;\mathrm{rate}\;(\%)=\frac{A_{0}-A_{1}}{A_{0}}\times 100$$

where *A*_0_ is the absorbance of the blank control, and *A*_1_ is the absorbance of the sample after addition of the composite film.

### 2.17. Antimicrobial Activity

The antibacterial and antifungal activities of the films were evaluated by the plate counting method against *Escherichia coli*, *Staphylococcus aureus*, and *Aspergillus niger*. Film samples were cut into 2 cm × 2 cm squares and sterilized by UV irradiation for 30 min before use. Seven treatments were examined: control, KGM, KGM/CMC film (KC), KGM/CMC/PVA film (KCP), KCP-HH1, KCP-HH2, and KCP-HH3. For the antibacterial assay, each film sample was placed in a 24-well plate and completely immersed in a bacterial suspension of *E. coli* or *S. aureus* (1 × 10^6^ CFU/mL). The plates were incubated at 37 °C with shaking for 24 h. The recovered bacterial suspensions were serially diluted tenfold with sterile phosphate-buffered saline (PBS), and 100 μL aliquots of appropriate dilutions were spread onto LB agar plates. After incubation at 37 °C for 24 h, the colonies were counted.

The antifungal assay was performed according to the method described by Yurtsever et al. [16], with minor modifications. Potato dextrose broth (PDB) and potato dextrose agar (PDA) were prepared according to the manufacturer’s instructions and sterilized before use. A spore suspension of A. niger was prepared and diluted to 1 × 10^5^ spores/mL using 1/500 PDB. A 64 μL aliquot of the diluted spore suspension was evenly applied to each film sample. The inoculated surface was covered with a sterile 1.6 cm × 1.6 cm cover film to ensure uniform contact between the spores and the test film. After static incubation at 28 °C for 24 h, spores were recovered from each sample using 6.4 mL of sterile PBS. The eluates were serially diluted tenfold, and 100 μL aliquots were spread onto PDA plates. The plates were incubated at 28 °C for 48 h before colony counting.

The antimicrobial inhibition rates were calculated as follows:

(9) $$I\mathrm{nhibition}\;\mathrm{rate}\;(\%)=\frac{N_{0}-N_{1}}{N_{0}}\times 100$$

where *N*_0_ is the colony count of the control group, and *N*_1_ is the colony count of the group treated with each film sample.

### 2.18. Soil Degradation Performance

The soil degradability of the films was evaluated using a modified soil burial test based on a previously reported method [14]. KGM, KC, KCP, KCP-HH1, KCP-HH2, and KCP-HH3 films were buried in natural soil, with polyethylene (PE) film used as the control. The soil was maintained at 25 °C and 60% of its water-holding capacity under aerobic conditions throughout the test. The visual appearance changes of the films were recorded using a digital camera on days 0, 7, 15, 23, and 30.

### 2.19. Strawberry Preservation Application

PE, KGM, KC, KCP, KCP-HH1, KCP-HH2, and KCP-HH3 films were cut into appropriate sizes. Strawberries were weighed and placed into polypropylene preservation boxes. Openings of identical size were made in the center of the box lids, which were then covered and fixed with the corresponding films. The packaged strawberries were stored at 25 ± 2 °C and 60 ± 5% relative humidity (RH). The appearance changes of the strawberries were recorded on days 0, 3, 6, 9, and 12 of storage, and their firmness, weight loss, total soluble solids (TSS), and pH were measured.

### 2.20. Appearance Evaluation

The appearance of the seven groups of strawberries was photographed on days 0, 3, 6, 9, and 12.

### 2.21. Firmness Measurement

Strawberry firmness was measured using a texture analyzer (TA.XT Plus, Stable Micro Systems Ltd., Godalming, UK) with a 2 mm diameter P/2 probe. For each fruit, measurements were performed at two opposite points in the equatorial region, avoiding the calyx and tip regions. The penetration depth was set at 5 mm. The pre-test speed, test speed, and post-test speed were set to 5.0, 1.0, and 1.0 mm/s, respectively. Each group was tested three times.

### 2.22. Weight Loss Measurement

The strawberries were weighed during storage using an electronic balance, and the weight loss rate was calculated according to the following equation:

(10) $$Weight\;loss\;(\%)=\frac{W_{0}-W_{i}}{W_{0}}\times 100$$

where *W*_0_ is the initial weight of the strawberries on day 0, and *W_i_* is the weight of the strawberries on days 3, 6, 9, and 12.

### 2.23. Total Soluble Solids Measurement

The strawberry juice was measured using a refractometer (PAL-1, ATAGO Co., Ltd., Tokyo, Japan) to obtain the TSS content on days 0, 3, 6, 9, and 12.

### 2.24. pH Measurement

Strawberry juice was extracted and used to determine the pH values on days 0, 3, 6, 9, and 12 using a pH meter (FE28-Standard, Mettler-Toledo Instruments Co., Ltd., Shanghai, China).

### 2.25. Statistical Analysis

All data are expressed as the mean ± standard deviation (M ± SD). Mechanical property measurements were performed in five replicates, while all other experiments were performed in triplicate unless otherwise stated. Statistical analysis was conducted using SPSS 26.0 (IBM Corp., Armonk, NY, USA), and figures were prepared using Origin 2022 (OriginLab Corporation, Northampton, MA, USA). We performed one-way ANOVA to evaluate intergroup differences, followed by Tukey’s multiple comparison test. with statistical significance set at *p* < 0.05. Different lowercase letters in the figures indicate significant differences among groups (*p* < 0.05).

## 3. Results and Discussion

### 3.1. Characterization of the HP-β-CD/HES Inclusion Complex and Films

#### 3.1.1. Encapsulation Efficiency and Zeta Potential of the HP-β-CD/HES Inclusion Complex

**Figure 1:**
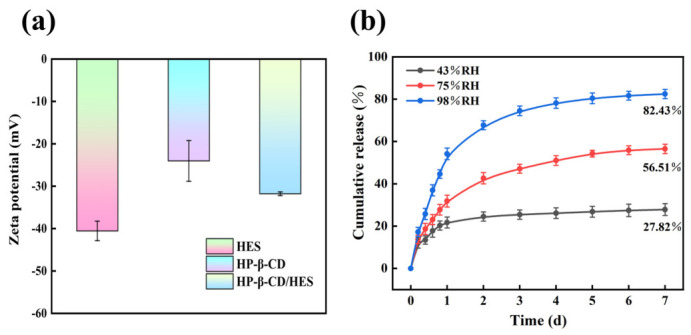
Zeta potential of HES, HP-β-CD, and HP-β-CD/HES (**a**); cumulative release behavior of HES from KCP-HH3 films under different relative humidity conditions (**b**).

The encapsulation efficiency of the HP-β-CD/HES inclusion complex reached 70.41 ± 4.01%, indicating that HP-β-CD exhibited a good inclusion capacity for HES. As shown in Figure 1a, the zeta potentials of HES, HP-β-CD, and the HP-β-CD/HES inclusion complex were −40.55 ± 2.30, −24.03 ± 4.81, and −31.78 ± 0.44 mV, respectively. Zeta potential is commonly used to evaluate dispersion stability, and an absolute value of approximately 30 mV or higher is generally associated with good colloidal stability [17,18]. Compared with HP-β-CD, the inclusion complex showed a higher absolute zeta potential value, suggesting enhanced electrostatic repulsion between particles and improved dispersion stability.

#### 3.1.2. Release Behavior of HES from KCP-HH3 Films

The sustained release behavior of HES from KCP-HH3 films under different relative humidity (RH) conditions is shown in Figure 1b. The cumulative release of HES increased with RH, reaching 27.82 ± 2.82%, 56.52 ± 2.22%, and 82.43 ± 2.19% after 7 days at 43%, 75%, and 98% RH, respectively. High humidity also promoted the early release of HES. At 98% RH, 54.16 ± 2.76% of HES was released within 1 day, whereas the corresponding values at 43% and 75% RH were only 21.70 ± 2.57% and 31.82 ± 2.79%, respectively. Subsequently, the release rate decreased gradually and approached a plateau, indicating that the initial release of surface-accessible HES was followed by the slower migration of HES retained within the film matrix [12].

The above results can be mainly explained by the hydrophilic characteristics of the KGM/CMC/PVA composite matrix. KGM, CMC, and PVA contain abundant hydrophilic groups, such as hydroxyl and carboxyl groups, which favor water adsorption under humid conditions [7]. At higher RH, the film matrix could absorb more moisture and swell to a greater extent, increasing the contact between water molecules and the embedded HP-β-CD/HES inclusion complex and thereby facilitating HES migration from the film. Therefore, the limited water uptake at 43% RH resulted in a slower release, whereas the higher moisture availability at 75% and especially 98% RH led to a more pronounced release. The HP-β-CD/HES inclusion complex may also be involved in the sustained release of HES. Cyclodextrins possess a hydrophilic exterior and a relatively hydrophobic cavity, which enables them to accommodate hydrophobic guest molecules and improve their dispersion or retention within polymer systems [19]. In the KCP-HH3 film, HP-β-CD may partially retain HES in the film matrix, while moisture absorption and swelling gradually promote its outward migration. Thus, the release of HES from KCP-HH3 films was likely controlled by the combined effects of moisture uptake, matrix swelling, and HP-β-CD-assisted retention. This sustained release behavior may be advantageous for fresh fruit packaging, where a humid storage environment can promote the availability of active compounds during preservation.

#### 3.1.3. Microstructural Morphology of Composite Films

**Figure 2:**
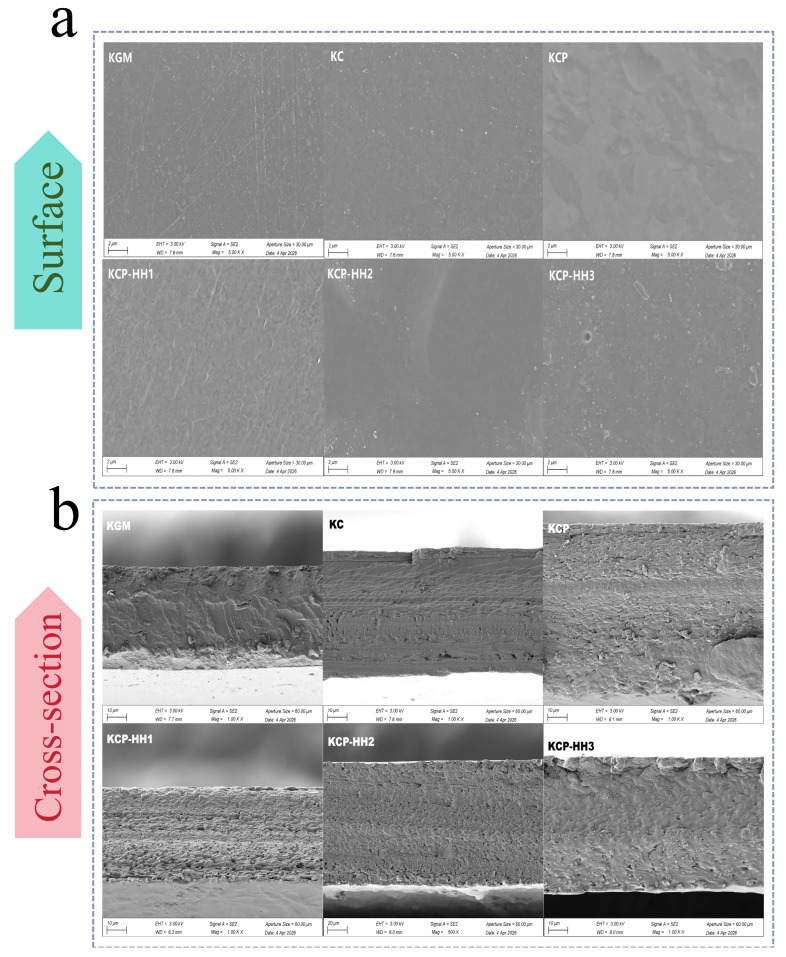
SEM images of the surface (**a**) and cross-section (**b**) of KGM, KC, KCP, KCP-HH1, KCP-HH2, and KCP-HH3 films.

As shown in Figure 2a,b, the pure KGM film exhibited a relatively rough surface and cross-section with slight aggregation, which is consistent with previous findings [20]. After CMC incorporation, the KC film showed a smoother surface and a more continuous and compact cross-section. This improvement may be associated with intermolecular interactions between KGM and the carboxyl and hydroxyl groups of CMC, which may have contributed to the improved compatibility and compactness of the film matrix. After further addition of PVA, the KCP film maintained good structural continuity, although slight irregular surface textures were observed. These morphological features may be associated with the participation of PVA in the composite network and its possible effect on structural rearrangement within the KGM/CMC matrix, thereby contributing to the continuity and stability of the film [21]. The incorporation of the HP-β-CD/HES inclusion complex further affected the film morphology. KCP-HH1 and KCP-HH2 retained relatively smooth surfaces and compact cross-sections, which may be consistent with a relatively uniform distribution of the inclusion complex within the polymer network. In contrast, KCP-HH3 showed obvious granular protrusions, increased surface roughness, and a more heterogeneous cross-section, suggesting local aggregation at higher loading levels. Such aggregation may reduce the compatibility between the inclusion complex and the polymer matrix, disrupt the ordered arrangement of molecular chains, and weaken the uniformity of the film network [22]. However, the observed morphological differences may also be influenced by variations in drying behavior and solvent evaporation during film formation, which may arise from differences in the initial film-forming compositions. Similar behavior has been reported in other active packaging films, where excessive incorporation of active substances increased surface roughness and structural heterogeneity [23].

#### 3.1.4. Chemical Structure Analysis of the HP-β-CD/HES Inclusion Complex and Composite Films

**Figure 3:**
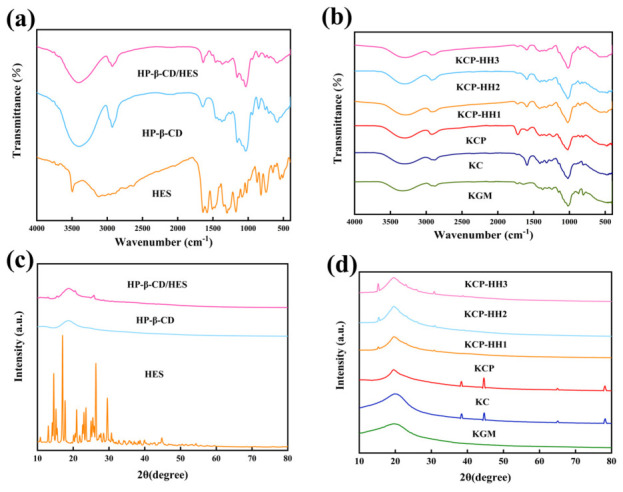
FT-IR spectra of HES, HP-β-CD, and HP-β-CD/HES inclusion complex (**a**); FT-IR spectra of KGM, KC, KCP, KCP-HH1, KCP-HH2, and KCP-HH3 films (**b**); XRD patterns of HES, HP-β-CD, and HP-β-CD/HES inclusion complex (**c**); XRD patterns of KGM, KC, KCP, KCP-HH1, KCP-HH2, and KCP-HH3 films (**d**).

As shown in Figure 3a, HES exhibited characteristic absorption peaks at 3495, 3116, 1581 and 1173 cm^−1^, corresponding to O–H stretching, aromatic =C–H stretching, C=C stretching of the benzene ring, and C–O/C–O–C stretching vibrations, respectively. HP-β-CD showed typical peaks at 3393, 2929, 1644, and 1033 cm^−1^, which were assigned to O–H stretching, C–H stretching, H–O–H bending of bound water, and C–O/C–O–C stretching vibrations, respectively. After complexation, the O–H stretching band of HP-β-CD shifted from 3393 to 3398 cm^−1^, while the peak at 1644 cm^−1^ shifted to 1637 cm^−1^. Meanwhile, the characteristic peaks of HES at 3116, 1581, and 1173 cm^−1^ were markedly weakened or disappeared. These spectral changes indicate that HES interacted with HP-β-CD through intermolecular forces, which altered the vibrational environment of the related functional groups and confirmed the formation of the HP-β-CD/HES inclusion complex. This inclusion process may be mainly attributed to van der Waals forces, dipole interactions, and hydrophobic interactions between the host and guest molecules [24].

As shown in Figure 3b, the KGM film displayed characteristic absorption bands at approximately 3432, 2920, and 1030 cm^−1^, corresponding to O–H stretching, C–H stretching, and C–O/C–O–C stretching vibrations, including those of glycosidic bonds, respectively [25]. After CMC incorporation, the KC film showed enhanced absorption near 1600 and 1410 cm^−1^, indicating the introduction of –COO^−^ groups from CMC. The subsequent addition of PVA caused broadening of the band around 3400 cm^−1^ and enhancement of the peak near 1030 cm^−1^, suggesting that PVA participated in the formation of the composite film network through hydrogen-bonding interactions with KGM and CMC [26]. Compared with KCP, no new characteristic peaks were observed in KCP-HH1, KCP-HH2, or KCP-HH3, indicating that no new covalent bonds were formed after adding the HP-β-CD/HES inclusion complex. However, the gradual changes in the bands around 3400, 1600, and 1030 cm^−1^ with increasing inclusion complex content suggest that the microenvironment of the original functional groups was altered. These results demonstrate that the inclusion complex was successfully incorporated into the film matrix and strengthened the non-covalent interactions among KGM, CMC, PVA, and HP-β-CD/HES.

#### 3.1.5. Crystalline Structure Analysis of HP-β-CD/HES Inclusion Complex and Composite Films

As shown in Figure 3c, HES exhibited distinct diffraction peaks at 2θ values of approximately 15.3°, 17.8°, 26.4°, and 29.5°, indicating its crystalline nature. In contrast, HP-β-CD showed a broad diffraction peak around 2θ = 20.2°, suggesting a predominantly amorphous structure. After complexation, the characteristic diffraction peaks of HES were markedly weakened or nearly disappeared, and the diffraction pattern of the HP-β-CD/HES inclusion complex changed to a broader amorphous profile. Only weak signals remained at approximately 2θ = 16.8°, 18.9°, and 22.1°, indicating that the ordered crystalline structure of HES was disrupted during inclusion. This reduction in crystallinity may be attributed to intermolecular interactions between HES and HP-β-CD, and is considered important evidence for the formation of cyclodextrin-based inclusion complexes [27].

As shown in Figure 3d, the KGM film displayed a broad diffraction peak at approximately 2θ = 20.8°, indicating its low crystallinity and mainly amorphous structure. The KC film retained a similar broad peak near 2θ = 21.1°, suggesting that CMC incorporation did not substantially change the crystalline characteristics of the KGM matrix. After PVA addition, the KCP film showed a stronger diffraction peak around 2θ = 20.2° with a slight shift to lower angles, indicating that hydrogen-bonding interactions among KGM, CMC, and PVA modified the molecular chain arrangement and slightly increased local structural order [26]. After further incorporation of the HP-β-CD/HES inclusion complex, KCP-HH1, KCP-HH2, and KCP-HH3 still exhibited broad diffraction peaks around 2θ = 19.6°, suggesting that the films remained predominantly amorphous. However, the sharper peak profiles indicated increased regularity of local chain segment arrangement [28]. Moreover, weak diffraction signals appeared near 2θ = 18.0° in the HP-β-CD/HES-loaded films, and their intensity gradually increased with increasing inclusion complex content. These results suggest that the inclusion complex was successfully incorporated into the film matrix and modified the internal structural organization of the composite films.

#### 3.1.6. Thermal Stability of HP-β-CD/HES Inclusion Complex and Composite Films

**Figure 4:**
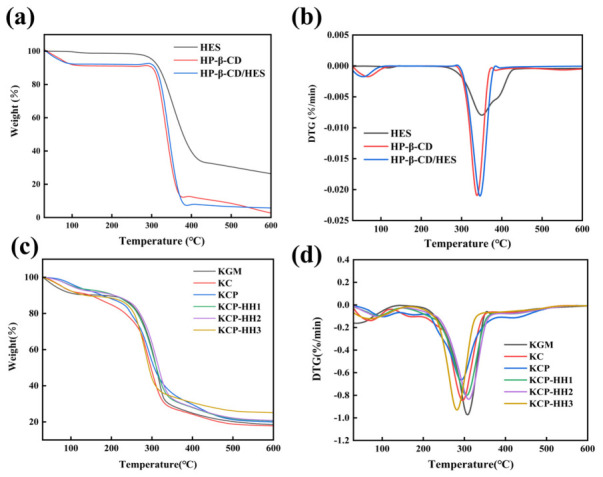
TG (**a**) and DTG (**b**) curves of HES, HP-β-CD, and the HP-β-CD/HES inclusion complex, and TG (**c**) and DTG (**d**) curves of KGM, KC, KCP, KCP-HH1, KCP-HH2, and KCP-HH3 films.

The TG and DTG curves in Figure 4a,b show that HES exhibited relatively high thermal stability, with its main decomposition starting at approximately 310 °C. No obvious weight loss was observed from room temperature to about 130 °C, which may be related to the hydrophobic nature of HES and its low affinity for water. In contrast, HP-β-CD showed slight weight loss below 100 °C due to the removal of bound water and volatile substances, followed by major degradation between 300 and 370 °C and slow decomposition from 380 to 600 °C. Notably, the thermal behavior of the HP-β-CD/HES inclusion complex was closer to that of HP-β-CD than to that of free HES, and no independent thermal decomposition peak of HES was observed, indicating that HES was successfully encapsulated within the HP-β-CD cavity.

For the composite films, the TG curves showed three main weight-loss stages (Figure 4c). The first stage, below approximately 130 °C, was mainly attributed to the evaporation of adsorbed water [29]. The second stage, occurring at 250–350 °C, corresponded to the main thermal degradation process, including dehydration, chain scission, and decomposition of polysaccharides and PVA [30]. The final stage, above 350 °C, was associated with carbonization and further decomposition of residual structures [31].

Compared with KGM, the KC and KCP films showed slightly higher decomposition temperatures, suggesting that CMC and PVA improved the thermal stability of the film matrix. After incorporation of the HP-β-CD/HES inclusion complex, the thermal degradation behavior of the KCP-HH films changed slightly. According to the DTG results in Figure 4d, the maximum weight-loss temperatures of KCP, KCP-HH1, and KCP-HH2 were 300, 305, and 311 °C, respectively, indicating that low inclusion complex levels of 0.25–0.5% did not adversely affect thermal stability. However, KCP-HH3 showed a lower maximum decomposition temperature of 292 °C, suggesting that excessive loading of the inclusion complex may disrupt the film network, accelerate thermal degradation, and slightly reduce thermal stability.

#### 3.1.7. Optical Properties and Transparency of Composite Films

**Figure 5:**
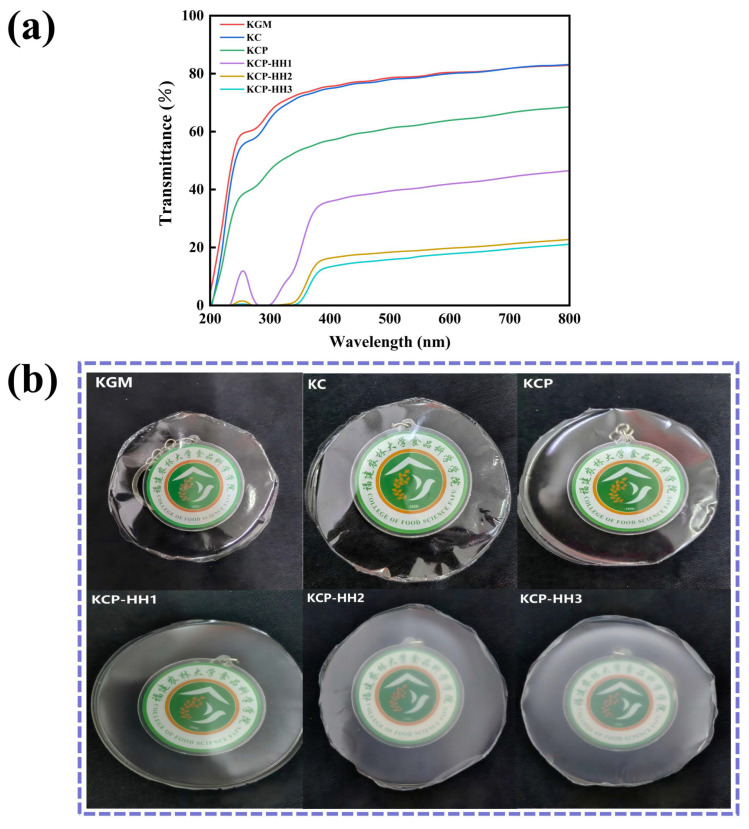
The UV–vis transmittance spectra (**a**), photographs showing the visual transparency of the corresponding composite films (**b**).

The UV–visible transmittance spectra of the composite films are presented in Figure 5a. KGM and KC films showed relatively high transmittance over the wavelength range of 200–800 nm, with similar spectral trends. In the ultraviolet region, their transmittance rapidly increased to above 50% and further increased in the visible region, reaching over 80% at approximately 800 nm. This indicates that KGM and KC had limited light-barrier capacity. Compared with these two films, KCP exhibited lower overall transmittance, with a value of approximately 69% at 800 nm. The reduced transparency may be attributed to stronger intermolecular interactions among KGM, CMC, and PVA, which promoted the formation of a denser film network and altered light transmission through the matrix [32].

After incorporation of the HP-β-CD/HES inclusion complex, the transmittance of the KCP-HH films decreased markedly. KCP-HH1 showed very low transmittance in the UV region and reached approximately 46% at 800 nm. KCP-HH2 and KCP-HH3 exhibited almost complete UV shielding in the range of 200–350 nm, and their transmittance values at 800 nm decreased to approximately 22% and 21%, respectively. These results demonstrate that HP-β-CD/HES effectively enhanced the light-barrier and UV-shielding properties of the composite films in a concentration-dependent manner. This improvement was mainly associated with HES, whose aromatic rings, conjugated structures, and phenolic hydroxyl groups can absorb UVA and UVB radiation, thereby reducing light transmission through the films [33]. The visual appearance of the films in Figure 5b was consistent with the transmittance results, showing a gradual decrease in transparency after the incorporation of HP-β-CD/HES, particularly in KCP-HH2 and KCP-HH3.

#### 3.1.8. Mechanical Properties of the Composite Films

**Figure 6:**
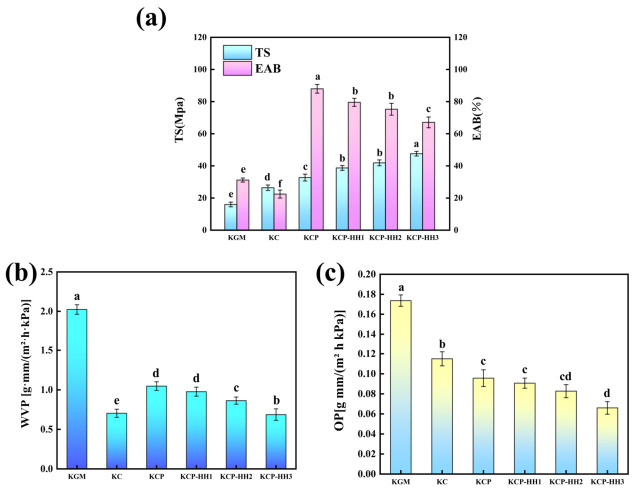
Mechanical properties (**a**), water vapor permeability (WVP) (**b**), and oxygen permeability (OP) (**c**) of KGM, KC, KCP, KCP-HH1, KCP-HH2, and KCP-HH3 films. Different lowercase letters above the bars indicate significant differences among different films within the same parameter (*p *< 0.05).

Mechanical properties are important indicators for evaluating the resistance of food packaging materials to external stress during handling, transportation, and storage. As shown in Figure 6a, the tensile strength (TS) and elongation at break (EAB) of the KGM film were 15.98 ± 1.45 MPa and 31.19 ± 1.27%, respectively. After CMC incorporation, the TS of the KC film increased to 26.39 ± 1.70 MPa, while the EAB decreased to 22.47 ± 2.48%. This result suggests that hydrogen-bonding interactions between KGM and CMC promoted a more compact molecular arrangement, thereby enhancing film rigidity but limiting polymer chain mobility and flexibility [34]. The addition of PVA further improved the mechanical performance of the composite film, increasing the TS and EAB of KCP to 32.73 ± 2.14 MPa and 87.98 ± 2.70%, respectively. This improvement may be attributed to stronger intermolecular interactions between PVA and the KGM/CMC matrix, which enhanced film strength. Meanwhile, the intrinsic flexibility and extensibility of PVA contributed to improved toughness and elasticity, resulting in a marked increase in EAB [35]. After incorporation of the HP-β-CD/HES inclusion complex, the TS of the KCP-HH films increased progressively with increasing inclusion complex content, whereas the EAB gradually decreased. This indicates that the inclusion complex reinforced the film matrix but reduced its flexibility. The enhancement in TS may be related to additional hydrogen bonding between the hydroxyl groups of HP-β-CD and the KGM, CMC, and PVA chains, as well as the reinforcing filler effect of the inclusion complex. However, higher loading levels restricted polymer chain mobility, leading to reduced flexibility and lower EAB values [36].

#### 3.1.9. Water Vapor Permeability of the Composite Films

Water vapor permeability (WVP) is a key parameter for evaluating the moisture barrier performance of food packaging films. Films with lower WVP can more effectively limit moisture transfer and help maintain food quality during storage. As shown in Figure 6b, the WVP values differed significantly among the film samples. The KGM film showed the highest WVP value of 2.02 ± 0.05 g·mm/(m^2^·h·kPa), indicating its weak moisture barrier capacity. This may be attributed to the relatively loose molecular structure and strong hydrophilicity of KGM, which facilitated water vapor diffusion through the film matrix [37]. After CMC incorporation, the WVP of the KC film decreased markedly to 0.70 ± 0.04 g·mm/(m^2^·h·kPa). This decrease suggests that hydrogen-bonding interactions between KGM and CMC improved the compactness of the film network, thereby extending the diffusion pathway of water molecules and reducing water vapor transmission. However, the addition of PVA increased the WVP of the KCP film to 1.05 ± 0.06 g·mm/(m^2^·h·kPa), which was likely due to the abundant polar hydroxyl groups in PVA that enhanced film hydrophilicity and promoted water vapor permeation [38]. With the incorporation of the HP-β-CD/HES inclusion complex, the WVP of the KCP-HH films gradually decreased. The WVP values of KCP-HH1, KCP-HH2, and KCP-HH3 were 0.98 ± 0.04, 0.86 ± 0.04, and 0.69 ± 0.06 g·mm/(m^2^·h·kPa), respectively. This improvement may be mainly attributed to the hydrophobic nature of HES, which increased the hydrophobicity of the composite films. In addition, intermolecular hydrogen bonding among HP-β-CD/HES, KGM, CMC, and PVA may have reduced the number of available hydrophilic groups and weakened the affinity of the film matrix for water molecules, thereby enhancing the moisture barrier performance of the KCP-based films [39].

#### 3.1.10. Oxygen Permeability of the Composite Films

Oxygen barrier performance is critical for food packaging films because it helps limit oxygen permeation, delay oxidative deterioration, and extend the shelf life of packaged foods. For films used in fruit and vegetable preservation, gas barrier properties are particularly important because they are closely related to the respiratory metabolism of fresh produce [40]. As shown in Figure 6c, the oxygen permeability (OP) values differed significantly among the film samples. The KGM film showed the highest OP value of 0.174 ± 0.006 g·mm/(m^2^·h·kPa), indicating weak oxygen barrier performance. This may be attributed to the relatively loose internal structure caused by weak intermolecular interactions among KGM chains, which allowed oxygen to diffuse more readily through the film matrix. This trend was consistent with the WVP results. After CMC incorporation, the OP of the KC film decreased to 0.115 ± 0.007 g·mm/(m^2^·h·kPa), suggesting that improved compatibility and stronger intermolecular interactions between KGM and CMC enhanced the compactness of the film network. Further addition of PVA reduced the OP of KCP to 0.096 ± 0.008 g·mm/(m^2^·h·kPa), which may be related to the formation of a denser KGM/CMC/PVA network through hydrogen bonding and other intermolecular interactions, thereby restricting oxygen diffusion [41]. The incorporation of the HP-β-CD/HES inclusion complex further decreased the OP of the KCP-HH films. The OP values of KCP-HH1, KCP-HH2, and KCP-HH3 were 0.091 ± 0.005, 0.083 ± 0.007, and 0.066 ± 0.006 g·mm/(m^2^·h·kPa), respectively, with KCP-HH3 showing the best oxygen barrier performance. This improvement may be attributed to strengthened hydrogen-bonding interactions among KGM, CMC, PVA, and HP-β-CD/HES, which promoted a more compact polymer chain arrangement. In addition, the inclusion complex may have partially filled the voids within the film network, further suppressing oxygen permeation [42].

#### 3.1.11. Antioxidant Properties of the Composite Films

**Figure 7:**
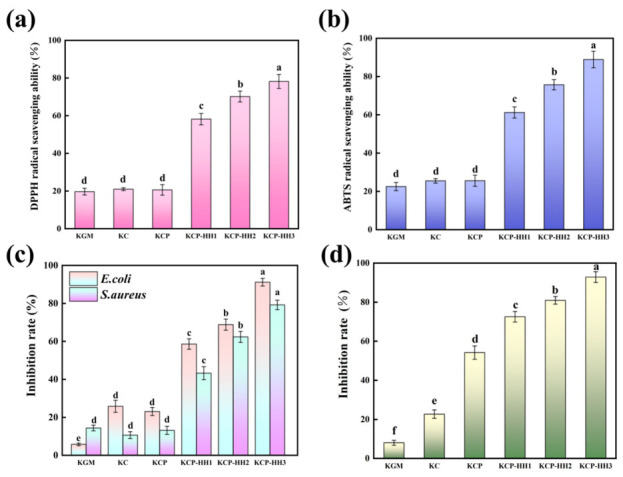
The DPPH radical-scavenging activity (**a**), ABTS radical-scavenging activity (**b**), inhibition rates against *E. coli* and *S. aureus* (**c**) and inhibition rate against *A. niger* (**d**) of KGM, KC, KCP, KCP-HH1, KCP-HH2, and KCP-HH3 films. Different lowercase letters above the bars indicate significant differences among different films within the same parameter (*p *< 0.05).

Antioxidant packaging can inhibit oxidative reactions in food, reduce nutrient and flavor deterioration, and extend product shelf life. Therefore, the antioxidant capacity of packaging materials is important for food preservation [43,44]. As shown in Figure 7a, the DPPH radical-scavenging rates of KGM, KC, and KCP films were relatively low, indicating weak intrinsic antioxidant activity in these film systems. This may be attributed to their limited ability to donate hydrogen atoms or electrons, resulting in insufficient scavenging activity against DPPH radicals [45]. After incorporation of the HP-β-CD/HES inclusion complex, the DPPH radical-scavenging activity of the composite films increased significantly. The DPPH radical-scavenging rates of KCP-HH1, KCP-HH2, and KCP-HH3 films were 58.17 ± 3.01%, 70.17 ± 2.88%, and 78.19 ± 3.71%, respectively, showing a gradual increase with increasing inclusion complex content. This enhancement was mainly attributed to HES, a flavonoid compound rich in phenolic hydroxyl groups, which can effectively neutralize free radicals by donating hydrogen atoms or electrons and thus exhibits strong antioxidant activity [46,47].

A similar trend was observed in the ABTS assay. As shown in Figure 6b, the ABTS radical-scavenging rates of KGM, KC, and KCP films were 22.54 ± 2.12%, 25.50 ± 1.17%, and 25.57 ± 2.89%, respectively, indicating that these films also had relatively weak ABTS radical-scavenging ability. In contrast, after incorporation of the HP-β-CD/HES inclusion complex, the ABTS radical-scavenging activity increased significantly, reaching 61.24 ± 2.90%, 75.72 ± 2.68%, and 88.89 ± 4.30% for KCP-HH1, KCP-HH2, and KCP-HH3, respectively. Among these films, KCP-HH3 exhibited the highest ABTS radical-scavenging activity. Overall, the incorporation of the HP-β-CD/HES inclusion complex markedly improved the antioxidant properties of the KGM-based composite films, highlighting their potential for application in antioxidant active food packaging.

#### 3.1.12. Antimicrobial Properties of Composite Films

As shown in Figure 7c, the KGM, KC, and KCP films exhibited relatively weak antibacterial activity against *Escherichia coli* and *Staphylococcus aureus*. The inhibition rates of these films against E. coli were 5.77 ± 2.50%, 25.81 ± 3.15%, and 23.08 ± 2.13%, respectively, while those against *S. aureus* were 14.38 ± 1.50%, 10.63 ± 1.83%, and 13.13 ± 3.08%, respectively. These results indicate that the KGM/CMC/PVA matrix itself had limited antibacterial activity in the absence of the HP-β-CD/HES inclusion complex. After incorporation of the HP-β-CD/HES inclusion complex, After incorporation of the HP-β-CD/HES inclusion complex, the antibacterial activity of the KCP-HH films was significantly enhanced. The inhibition rates of KCP-HH1, KCP-HH2, and KCP-HH3 against *E. coli* increased to 58.60 ± 3.70%, 68.82 ± 2.90%, and 91.18 ± 2.99%, respectively, while those against *S. aureus* increased to 43.26 ± 3.39%, 62.36 ± 3.88%, and 79.21 ± 3.45%, respectively. The concentration-dependent increase in inhibition rates suggests that HP-β-CD/HES played a major role in improving the antibacterial performance of the composite films.

The antifungal activity of the composite films against *Aspergillus niger* is shown in Figure 7d. KGM and KC showed relatively low inhibition rates of 8.06 ± 1.28% and 22.71 ± 2.16%, respectively, whereas the inhibition rate of KCP increased to 54.21 ± 3.37%. This result suggests that the KCP composite matrix provided a stronger inhibitory effect against *A. niger* than the KGM or KC films. After loading with the HP-β-CD/HES inclusion complex, the antifungal activity further increased in a concentration-dependent manner. The inhibition rates of KCP-HH1, KCP-HH2, and KCP-HH3 against *A. niger* were 72.53 ± 2.49%, 80.95 ± 1.91%, and 92.85 ± 2.75%, respectively. Among all samples, KCP-HH3 exhibited the highest antifungal activity, which was significantly higher than that of the other films.

This enhancement was mainly attributed to HES, a bioactive flavonoid with intrinsic antibacterial activity against both *E. coli* and *S. aureus* [48]. Previous studies have also reported the antifungal potential of HES against several fungal strains, including *A. niger*. Flavonoids can inhibit bacterial growth by disrupting cell wall and membrane integrity, increasing membrane permeability, inducing leakage of intracellular proteins and nucleic acids, and interfering with microbial energy metabolism [49]. For fungi, flavonoid-related antifungal effects may involve damage to the fungal cell wall and plasma membrane, changes in membrane permeability, interference with mitochondrial function and energy metabolism, and oxidative-stress-related injury. In addition, encapsulation by HP-β-CD can improve the water solubility, dispersibility, and stability of HES, thereby promoting its more uniform distribution within the film matrix. This may facilitate the sustained release of HES during contact between the KCP-HH films and bacterial suspensions, resulting in improved antibacterial and antifungal efficiencies [50]. Therefore, the KCP-HH films show promising potential as antimicrobial active packaging materials for food preservation.

#### 3.1.13. Soil Degradability of the Composite Films

**Figure 8:**
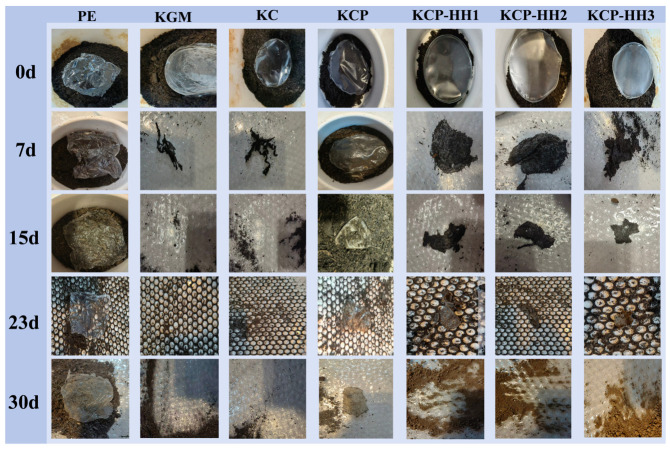
The biodegradability of PE, KGM, KC, KCP, KCP-HH1, KCP-HH2, and KCP-HH3 films in natural soil after 30 days.

The soil degradation behavior of composite films is an important indicator for evaluating their environmental compatibility as food packaging materials. Materials with good degradability can gradually lose structural integrity in biologically active environments, such as soil or compost, under the action of moisture, microorganisms, and enzymes [51]. As shown in Figure 8, the PE film maintained an intact morphology after 30 days, with no obvious signs of degradation. In contrast, no visible film residues of the KGM and KC films were observed after 15 days of soil burial. This rapid degradation may be attributed to the polysaccharide nature and high hydrophilicity of KGM and CMC, which make the films more susceptible to water absorption and microbial utilization. The absorbed moisture could also promote microbial adhesion and growth on the film surface, thereby accelerating structural disintegration [52]. Compared with KGM and KC, visible residues of the KCP film were still observed after 30 days of soil burial, suggesting that the incorporation of PVA may have delayed the structural disintegration of the film. This may be related to the relatively high crystallinity of PVA and its stable carbon-chain structure, which can increase resistance to direct microbial erosion and decomposition. After incorporation of the HP-β-CD/HES inclusion complex, the KCP-HH films were completely degraded within 30 days, and the degradation rate increased with increasing inclusion complex content. This behavior may be associated with changes in the internal structure and moisture sensitivity of the films. In particular, the hydrophilic HP-β-CD component may enhance water absorption and swelling of the composite films in soil, thereby facilitating microbial penetration and accelerating the degradation of the KCP-HH films.

### 3.2. Application of the Films in Strawberry Preservation

#### 3.2.1. Appearance Analysis

**Figure 9:**
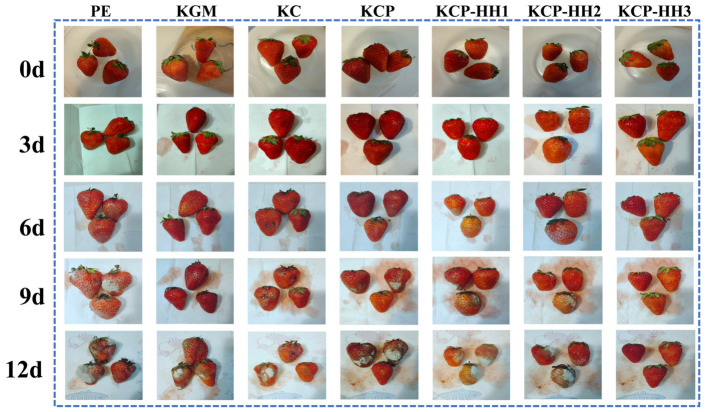
Appearance changes of strawberries packaged with PE, KGM, KC, KCP, KCP-HH1, KCP-HH2, and KCP-HH3 films during 12 days of storage.

As shown in Figure 9, the appearance quality of strawberries gradually deteriorated during storage, but the extent of deterioration varied among the packaging treatments. During the first 3 days, only slight differences were observed among the groups, and most strawberries showed no obvious visible decay during the first 3 days of storage. However, slight decay was already visible in the PE control group. By day 6, strawberries packaged with PE, KGM, and KC showed obvious spoilage and had largely lost marketability, whereas those treated with KCP and HP-β-CD/HES-loaded films still maintained better shape and color. At 9–12 days, quality differences became more pronounced. Severe softening, decay, black spots, and mold growth were observed in the PE, KGM, KC, and KCP groups, while the KCP-HH films more effectively delayed visible deterioration. Among the treatments, KCP-HH3 showed relatively better preservation performance, as the strawberries exhibited less visible deterioration and retained a more acceptable appearance after 12 days of storage. This effect may be associated with the improved barrier properties of the HP-β-CD/HES-loaded films, which could help regulate the package microenvironment and delay quality loss. In addition, the antioxidant and antimicrobial activities of HES may have contributed to reducing oxidative deterioration and microbial growth during storage.

#### 3.2.2. Texture Analysis

**Figure 10:**
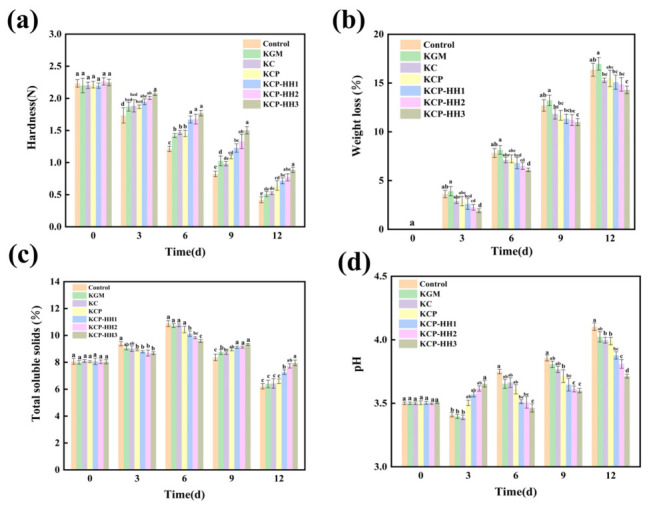
Changes in hardness (**a**), weight loss rate (**b**), total soluble solids (TSS) (**c**), and pH (**d**) of strawberries packaged with PE, KGM, KC, KCP, KCP-HH1, KCP-HH2, and KCP-HH3 films during 12 days of storage. Different lowercase letters above the bars indicate significant differences among different films within the same parameter (*p *< 0.05).

Firmness is an important indicator of postharvest textural quality in strawberries because it reflects fruit tissue integrity and storage tolerance [53]. During storage, the degradation of cell wall components can lead to tissue loosening and fruit softening, resulting in a gradual decline in firmness [54]. As shown in Figure 10a, the initial firmness of strawberries in all groups was approximately 2.20 ± 0.04 N, indicating comparable starting quality. Firmness decreased continuously during storage, but the rate of softening varied among the packaging treatments. After 12 days, the firmness of strawberries in the PE control group decreased to 0.42 ± 0.05 N, which was lower than that of the other groups. The KGM, KC, and KCP groups showed firmness values of 0.51 ± 0.04, 0.52 ± 0.02, and 0.64 ± 0.08 N, respectively, indicating that these films only partially delayed fruit softening. In contrast, the HP-β-CD/HES-loaded films better maintained strawberry firmness, with values of 0.72 ± 0.05, 0.77 ± 0.06, and 0.89 ± 0.04 N for KCP-HH1, KCP-HH2, and KCP-HH3, respectively. Among them, KCP-HH3 exhibited the highest firmness, suggesting the most effective inhibition of tissue softening. Similar preservation behavior has also been reported for KGM-based multifunctional packaging films used in strawberry preservation [55]. This effect may be attributed to the improved barrier, antioxidant, and antibacterial properties of the HP-β-CD/HES-loaded films, which helped reduce moisture loss, microbial decay, and metabolic deterioration during storage.

#### 3.2.3. Weight Loss Rate Analysis

Weight loss is an important indicator of water loss in strawberries during storage and can reflect the moisture-retention ability of packaging materials. Previous studies have shown that strawberry weight loss generally increases during storage, mainly due to transpiration and respiratory metabolism [56]. As shown in Figure 10b, the weight loss rate increased continuously in all groups during storage. On day 12, the weight loss rates of strawberries packaged with PE, KGM, KC, KCP, KCP-HH1, KCP-HH2, and KCP-HH3 were 16.36 ± 0.66%, 16.96 ± 0.64%, 15.29 ± 0.23%, 15.48 ± 0.84%, 15.09 ± 0.73%, 14.86 ± 0.70%, and 14.30 ± 0.40%, respectively. Among the treatments, KCP-HH3 showed the lowest weight loss rate, indicating the best ability to limit moisture loss. Although the PE group showed a lower weight loss rate than the KGM group, the KC, KCP, and KCP-HH groups generally showed improved moisture-retention effects compared with the KGM group. In particular, the weight loss rate decreased gradually with increasing HP-β-CD/HES inclusion complex content. Notably, the KCP-HH3 group showed a significantly lower weight loss rate than the PE group from day 3 to day 12 of storage. This trend was consistent with the WVP results, suggesting that the enhanced water vapor barrier properties of the HP-β-CD/HES-loaded films may have contributed to retard transpiration-induced water loss and maintaining strawberry quality during storage [57].

#### 3.2.4. Total Soluble Solids Analysis

Total soluble solids (TSS) are an important indicator of strawberry flavor quality and maturity, reflecting changes in soluble sugars and other soluble compounds in the fruit [58]. As shown in Figure 10c, the TSS content of strawberries in all groups initially increased and then decreased during storage, which is consistent with previous findings [59]. The increase in TSS during the early storage stage may be attributed to continued postharvest ripening, during which insoluble substances are converted into soluble sugars. Moderate water loss may also produce a concentration effect, further contributing to the increase in TSS [60]. As storage progressed, TSS began to decline, mainly due to the consumption of soluble sugars through respiration, accelerated senescence, and nutrient loss associated with spoilage. Tissue damage caused by mold growth may have further promoted the loss of soluble solids [61]. On day 12, the TSS values of strawberries in the PE, KGM, KC, KCP, KCP-HH1, KCP-HH2, and KCP-HH3 groups were 6.22 ± 0.20%, 6.39 ± 0.29%, 6.43 ± 0.36%, 6.59 ± 0.17%, 7.26 ± 0.18%, 7.74 ± 0.16%, and 7.96 ± 0.21%, respectively. Among all treatments, KCP-HH3 maintained the highest TSS value, indicating that HP-β-CD/HES-loaded composite films were more effective in reducing soluble solid loss during the later stage of storage. This effect may be related to their improved barrier, antioxidant, and antibacterial properties, which helped delay respiration, senescence, and microbial spoilage, thereby contributing to strawberry quality preservation and shelf-life extension.

#### 3.2.5. pH Analysis

pH is an important indicator of postharvest acidity changes in strawberries and can reflect organic acid metabolism and quality deterioration during storage [58]. As shown in Figure 10d, the pH values of strawberries in all groups generally increased during storage, but the extent of increase varied among the packaging treatments. The increase in pH may be attributed to the gradual consumption of organic acids during respiration and metabolic processes [62]. On day 12, the PE control group showed the greatest pH increase, indicating the weakest ability to maintain fruit quality. The KGM, KC, and KCP films partially delayed the increase in pH, but their effects were limited. In contrast, the HP-β-CD/HES-loaded films resulted in smaller pH changes, and this effect became more pronounced with increasing inclusion complex content. Among all treatments, KCP-HH3 showed the smallest pH variation, indicating the best performance in maintaining strawberry quality. This effect may be attributed to the improved barrier properties of the HP-β-CD/HES-loaded films, which helped regulate the package microenvironment and inhibit strawberry respiration, thereby reducing organic acid consumption. In addition, the antioxidant and antibacterial activities of HES may have alleviated tissue senescence and microbial decay during storage, further delaying acidity-related quality deterioration [63].

## 4. Conclusions

KGM/CMC/PVA active composite films loaded with an HP-β-CD/HES inclusion complex were successfully prepared by solution casting. The inclusion complex exhibited an encapsulation efficiency of 70.41 ± 4.01%. Of the formulations evaluated, KCP-HH3, containing the highest inclusion-complex level (1.5%), showed the most favorable overall performance. It provided nearly complete UV shielding from 200 to 350 nm, while its transmittance at 800 nm was approximately 21%. Relative to the KCP film, KCP-HH3 exhibited lower water vapor permeability (0.69 ± 0.06 g·mm/(m^2^·h·kPa)) and oxygen permeability (0.066 ± 0.006 g·mm/(m^2^·h·kPa)). Under 98% relative humidity, the cumulative release of HES from KCP-HH3 reached 82.43 ± 2.19% after 7 days. The film also scavenged 78.19 ± 3.71% of DPPH radicals and 88.89 ± 4.30% of ABTS radicals. Inhibition rates were 91.18 ± 2.99% against *Escherichia coli*, 79.21 ± 3.45% against *Staphylococcus aureus*, and 92.85 ± 2.75% against *Aspergillus niger*. KCP-HH3 also gave relatively better preservation results for strawberries. After 12 days of storage, the firmness and total soluble solids of strawberries packaged with KCP-HH3 were 0.89 ± 0.04 N and 7.96 ± 0.21%, while weight loss was limited to 14.30 ± 0.40%. In the polyethylene control, firmness decreased to 0.42 ± 0.05 N, total soluble solids to 6.22 ± 0.20%, and weight loss increased to 16.36 ± 0.66%. Taken together, these findings support the use of HP-β-CD/HES-loaded KCP films as biodegradable active packaging for strawberry preservation.

Several limitations should, however, be considered. The antioxidant, antibacterial, and antifungal assays were performed at a single test concentration; dose-response relationships and IC_50_ values were therefore not established. Free HES was not separately examined by UV–vis spectroscopy or tested for its stability under UV irradiation. A systematic comparison of free HES, HP-β-CD, and the HP-β-CD/HES inclusion complex would help clarify the contribution of complexation to UV protection. The strawberry evaluation did not include instrumental color measurements (L, a, b*, and ΔE) or microbiological indicators. Film density was also not determined. In addition, the present oxygen-permeability data provide a relative comparison among the developed films but do not by themselves establish suitability for wider food-packaging applications, which depend on the food matrix, package design, sealing performance, and storage conditions. Future studies should address these issues through color and microbiological analyses, density measurement, oxygen-transmission and package-headspace gas measurements under relevant storage conditions, comparison with commercial materials, and migration and sensory studies. Validation with other perishable fruits will also be needed before broader practical application can be assessed.

## Figures and Tables

**Table 1:** Composition and sample codes of the prepared composite films.

Films Sample	KGM (%, *w*/*v*)	CMC (%, *w*/*v*)	PVA (%, *w*/*v*)	HP-β-CD/HES Inclusion Complex (%, *w*/*v*)	Glycerol (%, *w*/*v*)	Citric Acid (wt%, Based on Total Dry Polymer Mass)
KGM	0.5	-	-	-	0.25	-
KC	0.5	0.5	-	-	0.25	-
KCP	0.5	0.5	0.8	-	0.25	10
KCP-HH1	0.5	0.5	0.8	0.5	0.25	10
KCP-HH2	0.5	0.5	0.8	1	0.25	10
KCP-HH3	0.5	0.5	0.8	1.5	0.25	10

## Data Availability

The original contributions presented in this study are included in the article. Further inquiries can be directed to the corresponding authors.

## Abbreviations

The following abbreviations are used in this manuscript:

KGM	Konjac glucomannan
CMC	Carboxymethyl cellulose
PVA	Polyvinyl alcohol
HES	Hesperetin
DPPH	2,2-Diphenyl-1-picrylhydrazyl
ABTS	2,2-azino-bis (3-ethylbenzothiazoline-6-sulfonic acid)
*E. coli*	*Escherichia coli*
*S. aureus*	*Staphylococcus aureus*
WVP	Water vapor permeability
EE	Encapsulation Efficiency
OP	Oxygen permeability
EAB	Elongation at break
SEM	Scanning Electron Microscopy
TS	Tensile strength
XRD	X-ray Diffraction
FTIR	Fourier Transform Infrared Spectroscopy
TG	Thermo-Gravimetric
DTG	Derivative thermogravimetry
TSS	Total soluble solids
KC	KGM/CMC film
KCP	KGM/CMC/PVA film
PE	polyethylene film
